# Sex-specific associations of urinary N-acetyl-*β*-D-glucosaminidase with arterial stiffness

**DOI:** 10.3389/fmed.2026.1816283

**Published:** 2026-09-16

**Authors:** Qiong Cheng, Yu-Ting Huang, Wei Chen, Yen-Jen Chen, Wen-Cheng Li

**Affiliations:** 1 Department of Nephrology, Xiamen Chang-Gung Hospital, Xiamen, China; 2 Department of Medical Education, Chang Gung Memorial Hospital, at Linkou, Taoyuan, Taiwan; 3 Department of Family Medicine, Chang-Gung Memorial Hospital at Linkou, Taoyuan, Taiwan; 4 College of Medicine, Chang Gung University, Taoyuan, Taiwan

**Keywords:** brachial-ankle pulse wave velocity, Cardiometabolic risk factors, endothelial dysfunction, gender differences in cardiovascular disease, renal tubular biomarkers, vascular aging

## Abstract

**Introduction:**

Urinary N-acetyl-β-D-glucosaminidase (uNAG), a sensitive biomarker of renal tubular injury, has been linked to vascular pathology through oxidative stress, inflammation, and endothelial dysfunction. However, sex-specific associations between uNAG and early vascular damage remain unclear.

**Methods:**

This cross-sectional study included 2,621 adults undergoing routine health examinations. Arterial stiffness was assessed using brachial-ankle pulse wave velocity (baPWV), with increased arterial stiffness defined as a mean baPWV >1,700 cm/s. uNAG and baPWV were measured using standardized protocols. Sex-stratified logistic regression analyses were performed.

**Results:**

In men, participants in the highest uNAG tertile had more than twofold higher adjusted odds of increased arterial stiffness after adjustment for age, mean arterial pressure, antihypertensive medication use, fasting glucose, triglycerides, and LDL–C (OR, 2.372; 95% CI, 1.603–3.510; *P* = 1.55 × 10^−5^). In women, the highest tertile was not significantly associated with increased arterial stiffness (OR, 0.842; 95% CI, 0.528–1.342; *P* = 0.470). Women in the intermediate tertile had lower adjusted odds (OR, 0.570; 95% CI, 0.343–0.947; *P* = 0.030), although this finding warrants caution because female uNAG values substantially clustered at 5.00 IU/L. The sex–by–uNAG tertile interaction was significant (P for interaction = 0.003).

**Discussion:**

Elevated uNAG was more strongly associated with increased arterial stiffness in men than in women, suggesting sex-related effect modification. Because of the cross-sectional design, these findings indicate associations rather than causal relationships.

## 1 Introduction

N-acetyl-*β*-D-glucosaminidase (NAG) is a lysosomal enzyme primarily located in renal proximal tubular epithelial cells and is excreted in the urine upon tubular injury. As a sensitive marker of renal tubular dysfunction, urinary Nacetyl-β-D-glucosaminidase (uNAG) has been widely studied not only in nephrology but also in the context of systemic vascular and metabolic diseases. Although creatinine and estimated glomerular filtration rate (eGFR) are commonly used markers for renal function, they mainly reflect glomerular filtration and typically rise in late-stage kidney disease. In contrast, uNAG, along with other tubular markers such as kidney injury molecule-1 (KIM-1) and neutrophil gelatinase-associated lipocalin (NGAL), has been proposed as a more sensitive indicator of early renal tubular injury and may serve as an early warning signal for systemic vascular damage. Recent studies suggest that elevated uNAG levels may reflect early endothelial damage, oxidative stress, and inflammation, thereby linking it to cardiovascular and cerebrovascular pathophysiology (1, 2). Elevated uNAG levels have been associated with endothelial dysfunction, oxidative stress, inflammation, diabetes, hypertension, and chronic kidney disease (CKD), all of which are also linked to vascular remodeling and arterial stiffening (2).

Arterial stiffness is an important feature of vascular aging and arteriosclerotic vascular remodeling. It may develop before overt cardiovascular disease and is associated with an increased risk of cardiovascular and cerebrovascular events. Arterial stiffening reflects structural and functional changes in the arterial wall, including elastin fragmentation, collagen accumulation, vascular smooth muscle cell remodeling, inflammation, and endothelial dysfunction (2). These processes may also contribute to progressive renal injury, suggesting a close relationship between renal tubular stress and systemic vascular health (3).

Brachial-ankle pulse wave velocity (baPWV) is a validated, non-invasive measure of arterial stiffness. Elevated baPWV has been strongly associated with increased cardiovascular risk and has been shown to predict cardiovascular events in both healthy and at-risk populations (4, 5). It is also increasingly utilized in large-scale epidemiological screening due to its reproducibility and clinical utility.

Sex differences may play an important role in the relationship between renal tubular injury and vascular dysfunction. Men and women differ in renal physiology, body fat distribution, endothelial function, inflammatory responses, and the trajectory of vascular aging. Estrogen may exert vasoprotective effects through enhancement of nitric oxide bioavailability, attenuation of oxidative stress, and modulation of vascular smooth muscle cell function, whereas the decline in estrogen after menopause may accelerate arterial stiffening (6). In contrast, men often develop arterial stiffness and cardiometabolic risk accumulation earlier in adulthood, which may increase susceptibility to vascular injury associated with renal tubular stress. Therefore, the association between uNAG and arterial stiffness may differ by sex.

Given the shared pathophysiological pathways of renal tubular injury and arterial stiffening—including inflammation, oxidative stress, endothelial dysfunction, and metabolic dysregulation—limited research has investigated the relationship between uNAG and baPWV, particularly with respect to sex-specific associations. Based on these considerations, we hypothesized that the association between uNAG and arterial stiffness would be sex specific. The aim of this study was therefore to investigate the relationship between uNAG and baPWV, with a primary focus on whether this association differs between men and women. We also examined whether higher uNAG levels were independently associated with increased arterial stiffness, defined as a mean baPWV >1,700 cm/s.

## 2 Material and methods

### 2.1 Population

This was a cross-sectional observational study including adults aged ≥18 years who attended annual health examination at Xiamen Chang-Gung Hospital during 2016∼2017. The study was approved by the Institutional Review Board of Xiamen Chang-Gung Hospital and conducted in accordance with the guidelines laid down in the Declaration of Helsinki.

### 2.2 Inclusion criteria

Patients enrolled in our study had complete medical records including past medical history and drug history. During the physical examination, the subjects had fasted for more than 12 hours, and non-pregnant women were recruited. Health examination items should include height, weight, WC, blood pressure (BP), total cholesterol (TC), low-density lipoprotein cholesterol (LDL-C), high-density lipoprotein cholesterol (HDL-C), triglycerides (TG), fasting glucose, NAG, baPWV and ankle-brachial index (ABI).

### 2.3 Exclusion criteria

Patients who meet the following criteria were excluded from the research: (1) chronic diseases that could significantly impact metabolism, such as thyroid dysfunction or chronic hepatitis, (2) current use of hypoglycemic agents or corticosteroids.

### 2.4 Data collection and measurements

During the health examination, participants were surveyed regarding their medical history, including any previous diseases or medication usage. Trained nurses followed standard operating procedures (SOP) for collecting venous blood samples and administering questionnaires to gather data.

The clinical chemistry workup included various measurements, conducted following the standardized procedures of a hospital laboratory accredited by the College of American Pathologists (CAP). The fasting glucose, TC, LDL-C, HDL-C, and TG were measured using a biochemical autoanalyzer (DxC 800, Beckman Coulter UniCel DxCSYNCHRON, Ireland). The uNAG level was measured by a colorimetric method using a reagent from Nittobo Medial Co., Ltd. (Tokyo, Japan). Accurate measurements were ensured using calibrated instruments.

Urinary N-acetyl-*β*-D-glucosaminidase values were analyzed as recorded in the electronic laboratory database. Measurements below 5.00 IU/L were present in the dataset, and no analytic rule was applied to replace values below 5.00 IU/L with 5.00 IU/L. However, substantial heaping at exactly 5.00 IU/L was observed among women. Because the data were obtained retrospectively, the original instrument-level output, analyzer flags, detailed assay documentation, and laboratory reporting metadata required to independently verify the assay-level lower limit of quantification and determine the origin of this clustering were unavailable.

In addition, the participants' BP, height, weight, and WC were measured using calibrated instruments. BP was recorded three times using an automated sphygmomanometer, with measurements taken after the participant had been in a seated position for at least 15 minutes. The mean arterial pressure (MAP) was estimated using the equation: 2/3 × diastolic pressure + 1/3 × systolic pressure. Height and weight were measured with precision to the nearest 0.1 cm and 0.1 kg, respectively. Body mass index (BMI) was calculated by dividing the weight in kilograms by the square of the height in meters (kg/m^2^). These measurements provided important data for the evaluation of participants' physical health and metabolic status.

baPWV was measured by using an ABI-form device (VP1000, Colin Co. Ltd, Komaki, Japan) that enables simultaneous measurement of systolic BP and the pulse waves of the brachial and posterior tibial arteries on all four extremities. The average of left and right baPWV and ABI for each individual was calculated. After having rest for more than 5 minutes in the supine position, cuffs were wrapped around bilateral arms and ankles and connected to a plethysmographic sensor and Oscillo-metric pressure sensor. ABI was measured by the ankle SBP divided by the brachial SBP. Pressure waveforms were recorded using semiconductor pressure sensors to assess the transmission time between the initial rises in both the brachial and tibial arteries waves. The distance between sampling points of baPWV was estimated based on height. The baPWV was calculated using the formula (La-Lb)/Tba. La is the distance from the heart to the ankle, Lb is the distance from the heart to the brachium, and Tba is the time interval between the brachial and ankle waveforms. The above measurement was performed twice, and the average values of the left- and right-side assessments were used as a marker of arterial stiffness.

### 2.5 Definition of increased arterial stiffness

Increased arterial stiffness was defined as a mean baPWV higher than 1,700 cm/s.

### 2.6 Statistical analysis

Parametric continuous variables were expressed as mean ± standard deviation (SD), and categorical data as number (percentage). Between-sex comparisons used the independent-samples Student t-test for continuous variables and the Pearson chi-square or Fisher exact test for categorical variables, as appropriate. Differences across sex-specific uNAG tertiles were evaluated using one-way analysis of variance for continuous variables and the Pearson chi-square or Fisher–Freeman–Halton exact test for categorical variables. Pearson correlation coefficients were calculated separately in men and women. The association between sex-specific uNAG tertiles and increased arterial stiffness was examined using univariate and multivariable logistic regression analyses. Model 1 was unadjusted. Model 2 was adjusted for age, mean arterial pressure, and antihypertensive medication use. Model 3 was additionally adjusted for fasting glucose, triglycerides, and LDL-C. The lowest uNAG tertile was used as the reference category in all models. Because this was a retrospective cross-sectional analysis of an existing health examination database, all participants who met the predefined eligibility criteria during the study period were included, and no prospective sample-size calculation was performed. Sample-size adequacy was evaluated using the number of increased arterial stiffness events relative to the predictor degrees of freedom in the multivariable models. To formally examine whether sex modified the association between uNAG and increased arterial stiffness, a pooled multivariable logistic regression model was fitted with the main effects of sex and sex-specific uNAG tertile and their interaction terms. The model was adjusted for age, mean arterial pressure, antihypertensive medication use, fasting glucose, triglycerides, and LDL-C. The overall interaction was evaluated using a likelihood-ratio test comparing models with and without the sex-by-uNAG tertile interaction terms. A sensitivity analysis was also conducted using the interaction between sex and log-transformed continuous uNAG. Renal function parameters, including serum creatinine, estimated glomerular filtration rate, and albuminuria, were unavailable and could not be included. *P* values ≥0.001 were reported to three decimal places, whereas smaller values were presented in scientific notation. All analyses were conducted using SPSS version 26.0 (SPSS, Chicago, IL, USA). A two-sided *P* value <0.05 was considered statistically significant.

## 3 Results

### 3.1 Baseline characteristics of the study population by sex

The baseline characteristics of the study population stratified by sex are summarized in Table 1. A total of 2,621 participants were included, comprising 1,476 men and 1,145 women. Women were slightly older than men (57.98 ± 9.17 vs. 57.17 ± 9.83 years; *P* = 0.032). No significant sex differences were observed in BMI, mean arterial pressure, waist circumference, waist-to-height ratio, fasting glucose, or lipid levels. uNAG was higher in men than in women (8.78 ± 8.64 vs. 6.07 ± 4.25 IU/L; *P* = 4.60 × 10⁻^22^), and women had lower ABI values (1.13 ± 0.08 vs. 1.16 ± 0.09; *P* = 1.47 × 10⁻^12^). baPWV did not differ significantly by sex (1435.03 ± 304.87 vs. 1458.05 ± 312.89 cm/s; *P* = 0.059). The overall prevalence of increased arterial stiffness was 14.88%, with no significant difference between men and women (14.36% vs. 15.55%; *P* = 0.399).

**Table 1:** Baseline characteristics of the study population by sex.

Characteristics	Total	Men	Women	*P* value
Number of subjects	2621	1476	1145	
Age, years	57.52 ± 9.56	57.17 ± 9.83	57.98 ± 9.17	0.032
BMI (kg/m^2^)	23.53 ± 3.11	23.57 ± 3.09	23.47 ± 3.14	0.444
Mean arterial pressure (mmHg)	88.07 ± 13.41	88.12 ± 13.57	88.02 ± 13.21	0.850
Waist circumference (cm)	82.21 ± 9.44	82.26 ± 9.27	82.15 ± 9.65	0.773
Waist-to-height ratio (cm/cm)	0.50 ± 0.06	0.50 ± 0.05	0.50 ± 0.06	0.672
Fasting glucose (mmol/L)	5.19 ± 1.33	5.18 ± 1.27	5.19 ± 1.39	0.826
Total cholesterol (mmol/L)	5.15 ± 0.98	5.15 ± 0.93	5.15 ± 1.04	0.974
Triglycerides (mmol/L)	1.55 ± 1.42	1.55 ± 1.26	1.55 ± 1.60	0.883
LDL cholesterol (mmol/L)	3.50 ± 0.90	3.50 ± 0.88	3.50 ± 0.92	0.971
HDL cholesterol (mmol/L)	1.31 ± 0.32	1.31 ± 0.31	1.31 ± 0.33	0.884
uNAG (IU/L)	7.59 ± 7.19	8.78 ± 8.64	6.07 ± 4.25	4.60 × 10⁻^22^
baPWV (cm/s)	1447.99 ± 309.56	1458.05 ± 312.89	1435.03 ± 304.87	0.059
ABI	1.15 ± 0.08	1.16 ± 0.09	1.13 ± 0.08	1.47 × 10⁻^12^
IAS	390 (14.88%)	212 (14.36%)	178 (15.55%)	0.399

BMI, body mass index; LDL, low-density lipoprotein; HDL, high-density lipoprotein; NAG, N-acetyl-*β*-D-glucosaminidase; PWV, pulse wave velocity; ABI, ankle-brachial index; IAS, increased arterial stiffness.

### 3.2 Pearson correlation coefficients between uNAG and cardiometabolic risk factors, stratified by sex

The correlations between uNAG and cardiometabolic risk factors stratified by sex are shown in Table 2 and Figure 1. uNAG was positively correlated with baPWV in both men and women, although the magnitude of the correlations was modest. The correlation was stronger in men (r = 0.240; *P* = 9.76 × 10⁻^21^) than in women (r = 0.102; *P* = 5.41 × 10⁻⁴). In women, uNAG showed stronger correlations with adiposity markers, including waist circumference (r = 0.212; *P* = 4.66 × 10⁻^13^), and was inversely correlated with HDL-C (r = −0.123; *P* = 2.86 × 10⁻⁵). Complete correlation results are presented in Table 2.

**Table 2:** Pearson correlation coefficients between uNAG and cardiometabolic or vascular factors, stratified by sex.

Characteristics	Men		Women	
	r	P value	r	*P* value
Age (year)	0.098	1.65 × 10⁻⁴	0.160	5.41 × 10⁻⁸
BMI (kg/m2)	0.094	3.21 × 10⁻⁴	0.222	3.03 × 10⁻^1^⁴
Mean arterial pressure (mmHg)	0.060	0.021	0.090	0.002
Waist circumference (cm)	0.096	2.30 × 10⁻⁴	0.212	4.66 × 10⁻^13^
Waist-to-height ratio (cm/cm)	0.064	0.014	0.166	1.50 × 10⁻⁸
Fasting glucose (mmol/L)	0.050	0.057	−0.003	0.925
Total cholesterol (mmol/L)	0.093	3.42 × 10⁻⁴	−0.035	0.234
Triglycerides (mmol/L)	0.039	0.137	0.042	0.159
LDL cholesterol (mmol/L)	0.084	0.001	−0.014	0.644
HDL cholesterol (mmol/L)	−0.006	0.825	−0.123	2.86 × 10⁻⁵
baPWV (cm/s)	0.240	9.76 × 10⁻^21^	0.102	5.41 × 10⁻⁴
ABI	−0.047	0.069	0.022	0.463

LDL, low-density lipoprotein; HDL, high-density lipoprotein; baPWV, brachial-ankle pulse wave velocity; ABI, ankle-brachial index.

**Figure 1:**
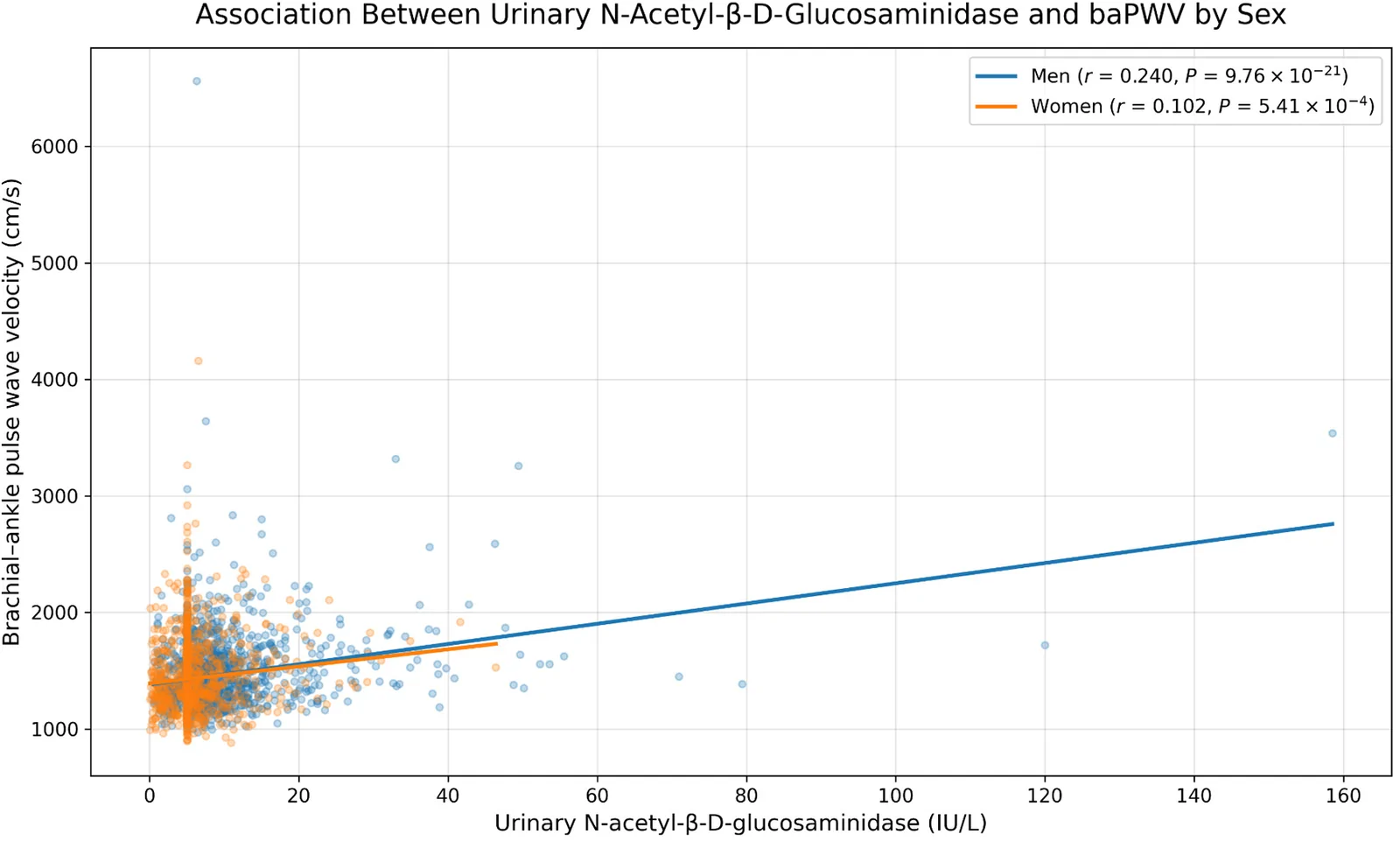
Association between urinary N-acetyl-*β*-D-glucosaminidase and brachial–ankle pulse wave velocity by sex. Scatter plots show individual uNAG and baPWV measurements in men and women. Solid lines represent sex-specific fitted linear regression lines. Pearson correlation analysis showed a positive correlation between uNAG and baPWV in men (r = 0.240; *P* = 9.76 × 10⁻^21^) and women (r = 0.102; *P* = 5.41 × 10⁻⁴).

### 3.3 Sex-Specific differences across tertiles of uNAG

Clinical characteristics across uNAG tertiles, stratified by sex, are presented in Tables 3, 4. Significant overall between-group differences were observed for several anthropometric, metabolic, and vascular variables; however, not all variables demonstrated monotonic changes across increasing uNAG tertiles. In men, age, triglycerides, uNAG, and baPWV generally increased from T1 to T3, whereas BMI, mean arterial pressure, waist circumference, waist-to-height ratio, fasting glucose, and HDL-C showed non-monotonic patterns. Men in T3 had the highest baPWV and prevalence of increased arterial stiffness compared with those in T1 (1529.83 ± 320.37 vs. 1411.39 ± 249.96 cm/s and 21.22% vs. 9.74%, respectively; *P* = 1.76 × 10⁻⁹ and *P* = 4.42 × 10⁻⁷, respectively).

**Table 3:** Clinical characteristics across sex-specific uNAG tertiles in men.

Sex	NAG			*P* value
Men	T1	T2	T3	
Number of subjects	493	493	490	
Age, years	56.20 ± 9.68	57.22 ± 9.80	58.11 ± 9.94	0.010
BMI (kg/m^2^)	22.44 ± 2.71	24.34 ± 3.08	23.93 ± 3.15	7.22 × 10⁻^2^⁴
Mean arterial pressure (mmHg)	85.59 ± 13.70	89.65 ± 12.78	89.12 ± 13.88	2.12 × 10⁻⁶
Waist circumference (cm)	78.67 ± 8.25	84.87 ± 9.16	83.23 ± 9.26	1.90 × 10⁻^2^⁷
Waist-to-height ratio (cm/cm)	0.49 ± 0.05	0.51 ± 0.05	0.51 ± 0.05	2.65 × 10⁻^13^
Fasting glucose (mmol/L)	5.03 ± 0.95	5.29 ± 1.41	5.22 ± 1.39	0.004
Total cholesterol (mmol/L)	5.12 ± 0.88	5.13 ± 0.94	5.20 ± 0.98	0.311
Triglycerides (mmol/L)	1.31 ± 0.81	1.65 ± 1.18	1.70 ± 1.62	7.69 × 10⁻⁷
LDL cholesterol (mmol/L)	3.45 ± 0.82	3.52 ± 0.87	3.53 ± 0.94	0.326
HDL cholesterol (mmol/L)	1.37 ± 0.31	1.26 ± 0.30	1.30 ± 0.32	1.66 × 10⁻⁸
uNAG (IU/L)	4.34 ± 1.17	6.32 ± 1.00	15.72 ± 12.18	7.28 × 10⁻^12^⁹
baPWV (cm/s)	1411.39 ± 249.96	1433.37 ± 347.99	1529.83 ± 320.37	1.76 × 10⁻⁹
ABI	1.16 ± 0.08	1.16 ± 0.09	1.15 ± 0.09	0.081
Antihypertensive medication use, n (%)	2 (0.41%)	5 (1.01%)	2 (0.41%)	0.528
Increased arterial stiffness, n (%)	48 (9.74%)	60 (12.17%)	104 (21.22%)	4.42 × 10⁻⁷

BMI, body mass index; LDL, low-density lipoprotein; HDL, high-density lipoprotein; NAG, N-acetyl-*β*-D-glucosaminidase; baPWV, pulse wave velocity; ABI, ankle-brachial index; IAS, increased arterial stiffness.

**Table 4:** Clinical characteristics across sex-specific uNAG tertiles in women.

Sex	NAG			*P* value
Women	T1	T2	T3	
Number of subjects	382	382	381	
Age, years	55.38 ± 8.53	58.98 ± 9.18	59.57 ± 9.26	4.97 × 10⁻^11^
BMI (kg/m^2^)	21.08 ± 2.28	25.20 ± 2.43	24.14 ± 3.05	5.80 × 10⁻⁹^3^
Mean arterial pressure (mmHg)	82.86 ± 12.53	91.24 ± 12.10	89.95 ± 13.42	8.18 × 10⁻^21^
Waist circumference (cm)	74.90 ± 7.79	87.56 ± 6.95	84.00 ± 9.24	2.69 × 10⁻⁹^1^
Waist-to-height ratio (cm/cm)	0.47 ± 0.05	0.53 ± 0.05	0.51 ± 0.05	2.82 × 10⁻⁴⁴
Fasting glucose (mmol/L)	4.94 ± 1.08	5.45 ± 1.77	5.18 ± 1.17	2.40 × 10⁻⁶
Total cholesterol (mmol/L)	4.97 ± 0.90	5.40 ± 1.20	5.07 ± 0.96	2.76 × 10⁻⁸
Triglycerides (mmol/L)	1.10 ± 0.62	1.99 ± 2.45	1.54 ± 0.97	6.96 × 10⁻^1^⁴
LDL cholesterol (mmol/L)	3.28 ± 0.84	3.75 ± 0.99	3.48 ± 0.87	7.55 × 10⁻^12^
HDL cholesterol (mmol/L)	1.46 ± 0.32	1.19 ± 0.28	1.27 ± 0.31	7.97 × 10⁻^33^
uNAG (IU/L)	3.499 ± 1.697	5.000 ± 0.005	9.713 ± 5.512	1.47 × 10⁻^122^
baPWV (cm/s)	1421.10 ± 281.85	1417.43 ± 306.76	1466.63 ± 322.95	0.046
ABI	1.13 ± 0.07	1.13 ± 0.08	1.14 ± 0.08	0.024
Antihypertensive medication use, n (%)	0 (0.00%)	2 (0.52%)	1 (0.26%)	0.554
Increased arterial stiffness, n (%)	55 (14.40%)	50 (13.09%)	73 (19.16%)	0.052

BMI, body mass index; LDL, low-density lipoprotein; HDL, high-density lipoprotein; NAG, N-acetyl-*β*-D-glucosaminidase; baPWV, pulse wave velocity; ABI, ankle-brachial index; IAS, increased arterial stiffness.

In women, age and uNAG increased across the groups, whereas most anthropometric and metabolic variables, including BMI, mean arterial pressure, waist circumference, fasting glucose, total cholesterol, triglycerides, and LDL-C, were highest in T2 rather than T3. HDL-C decreased from T1 to T2 and partially increased in T3. baPWV was similar in T1 and T2 but was higher in T3 (1421.10 ± 281.85, 1417.43 ± 306.76, and 1466.63 ± 322.95 cm/s, respectively; *P* = 0.046). The prevalence of increased arterial stiffness was 14.40%, 13.09%, and 19.16% in T1, T2, and T3, respectively, and the overall difference did not reach statistical significance (*P* = 0.052).

### 3.4 Unadjusted and multivariable-adjusted odds ratios for increased arterial stiffness

The unadjusted and multivariable-adjusted odds ratios for increased arterial stiffness according to uNAG tertiles are shown in Table 5. After adjustment for age, mean arterial pressure, antihypertensive medication use, fasting glucose, triglycerides, and LDL-C, men in the highest uNAG tertile had significantly higher odds of increased arterial stiffness than those in the lowest tertile (OR, 2.372; 95% CI, 1.603–3.510; *P* = 1.55 × 10⁻⁵). The intermediate tertile was not significantly associated with the outcome in men (OR, 1.189; 95% CI, 0.778–1.819; *P* = 0.424). In women, the highest uNAG tertile was not associated with increased arterial stiffness (OR, 0.842; 95% CI, 0.528–1.342; *P* = 0.470). The intermediate tertile was associated with lower adjusted odds (OR, 0.570; 95% CI, 0.343–0.947; *P* = 0.030), although this result should be interpreted cautiously because of the concentration of female uNAG measurements around 5.00 IU/L and the resulting sensitivity to tertile construction.

**Table 5:** Unadjusted and adjusted odds ratios (95% CIs) for increased arterial stiffness according to sex-specific uNAG tertiles.

	IAS									
NAG			Model 1			Model 2			Model 3	
	No. of events, n, (%)	OR	(95% CI)	P value	OR	(95% CI)	P value	OR	(95% CI)	*P* value
Men										
T1	48									
T2	60	1.285	0.859 to 1.920	0.222	1.176	0.770 to 1.796	0.454	1.189	0.778 to 1.819	0.424
T3	104	2.498	1.728 to 3.610	1.10 × 10⁻⁶	2.313	1.566 to 3.417	2.49 × 10⁻⁵	2.372	1.603 to 3.510	1.55 × 10⁻⁵
Women										
T1	55									
T2	50	0.895	0.593 to 1.352	0.599	0.497	0.304 to 0.814	0.005	0.570	0.343 to 0.947	0.030
T3	73	1.409	0.961 to 2.067	0.079	0.788	0.497 to 1.250	0.312	0.842	0.528 to 1.342	0.470

Model 1 was unadjusted. Model 2 was adjusted for age, mean arterial pressure, and antihypertensive medication use. Model 3 was additionally adjusted for fasting glucose, triglycerides, and LDL-C. T1 was the reference category. P for interaction: the overall interaction between sex and uNAG tertile was statistically significant in the fully adjusted pooled logistic regression model (likelihood-ratio *χ*^2^ = 11.58, 2 degrees of freedom; *P* = 0.003).

IAS, increased arterial stiffness; CI, confidence interval; OR, odds ratio.

The pooled multivariable model demonstrated a statistically significant overall interaction between sex and uNAG tertile for increased arterial stiffness (likelihood-ratio *χ*^2^ = 11.58, 2 degrees of freedom; *P* = 0.003). The ratio of odds ratios comparing men with women was 1.962 for T2 versus T1 (95% CI, 1.050–3.663; *P* = 0.035) and 2.710 for T3 versus T1 (95% CI, 1.518–4.836; *P* = 7.43 × 10⁻⁴), indicating a stronger positive association between higher uNAG categories and increased arterial stiffness in men. A sensitivity analysis using log-transformed continuous uNAG also showed a significant interaction with sex (P for interaction = 7.20 × 10⁻⁴).

## 4 Discussion

This study provides novel insights into the relationship between uNAG and arterial stiffness assessed by baPWV. The key findings are threefold. First, uNAG levels were significantly higher in men than in women and positively correlated with multiple cardiovascular risk factors including BMI, MAP, and baPWV. Second, elevated uNAG was independently associated with increased arterial stiffness in men, but not in women after multivariate adjustment, suggesting potential sex-specific pathophysiological pathways. Although uNAG was statistically significantly correlated with baPWV in both sexes, the magnitude of these correlations was modest. Therefore, the clinical relevance of uNAG as an individual marker of arterial stiffness should be interpreted with caution. The observed associations suggest that renal tubular injury may be linked to vascular stiffening; however, uNAG alone is unlikely to provide sufficient discriminatory value for clinical risk stratification without integration with established cardiovascular risk factors.

### 4.1 Adiposity, blood pressure, and NAG: comparisons and mechanisms

In this study, uNAG was positively associated with BMI, waist circumference, and mean arterial pressure in both sexes. These findings suggest that renal tubular injury may be related to obesity-associated hemodynamic stress and metabolic disturbances. The stronger correlations observed in women may reflect sex-related differences in body composition, renal physiology, cardiometabolic characteristics, or inflammatory responses. Aging may also modify these relationships. However, because menopausal status and sex hormone concentrations were not assessed, the contribution of hormonal changes cannot be determined from the present data.

### 4.2 Glucose, lipids, and uNAG: sex-specific patterns

The relationship between NAG and glucose or lipid profiles was less consistent. Fasting glucose and TG showed weak or no correlations with NAG in either sex, aligning with prior studies indicating that tubular markers such as NAG may precede overt metabolic derangement or reflect cumulative exposure rather than acute glycemic levels (7). In men, LDL-C exhibited a modest positive correlation, while in women, HDL-C showed an inverse relationship with NAG. The latter is biologically plausible, given HDL-C's anti-inflammatory and antioxidative properties that may mitigate tubular stress (8). Interestingly, TC and LDL-C differed across uNAG tertiles in women and were highest in T2, indicating variation in lipid profiles across the uNAG groups rather than a monotonic trend. These sex-related patterns may reflect differences in lipid metabolism, renal physiology, or other unmeasured factors; because menopausal status and sex hormone concentrations were unavailable, hormonal contributions could not be evaluated.

### 4.3 Arterial stiffness: uNAG as a vascular injury marker

One of the most important findings was that uNAG was positively correlated with baPWV in both sexes and was independently associated with increased arterial stiffness in men. Although the correlation between uNAG and baPWV in women was statistically significant, its magnitude was weak, suggesting limited clinical relevance. This aligns with emerging evidence suggesting that renal tubular injury and arterial stiffness may share common pathophysiological pathways, including systemic inflammation, oxidative stress, and endothelial dysfunction (4).

uNAG is a lysosomal enzyme released from proximal tubular epithelial cells in response to injury or oxidative stress. Its elevation is widely considered an early and sensitive biomarker of renal tubular damage, preceding overt changes in glomerular filtration rate (GFR) or albuminuria (9). Beyond the kidneys, recent research has highlighted the systemic implications of tubular injury, particularly its link to endothelial dysfunction and arterial stiffening. Chronic low-grade inflammation and oxidative stress—both common drivers of NAG elevation—are also known to impair nitric oxide bioavailability and promote vascular smooth muscle cell proliferation, key mechanisms underlying arterial stiffening (10, 11). Elevated uNAG levels are independently associated with increased arterial stiffness and cardiovascular risk, even after adjusting for renal function and traditional risk factors. Tanaka et al. reported that a higher urinary NAG activity-to-creatinine concentration ratio was associated with increased subsequent risks of disability and all-cause mortality in a community-based cohort, suggesting that uNAG may reflect adverse systemic outcomes beyond conventional renal measures (12). Consistent with this concept, Ouchi et al. reported that the urinary NAG-to-creatinine ratio was positively associated with baPWV in patients without diabetes, whereas Lee et al. found that an elevated urinary NAG-to-creatinine ratio was associated with increased carotid intima-media thickness in patients with type 1 diabetes, independently of albuminuria (2, 13). These findings suggest that renal tubular injury may be associated with both functional arterial stiffening and structural carotid vascular changes. Lou et al. further showed uNAG independently associated with carotid plaque in patients with type 2 diabetes (14). Our study extends these findings by examining the association between uNAG and baPWV in a broader, non-diabetic population, suggesting that uNAG may provide complementary information regarding arterial stiffness-related vascular changes (15). The sex-specific relationship between uNAG and arterial stiffness is further illustrated in Figure 1, while the adjusted odds ratios across uNAG tertiles are summarized in Figure 2.

**Figure 2:**
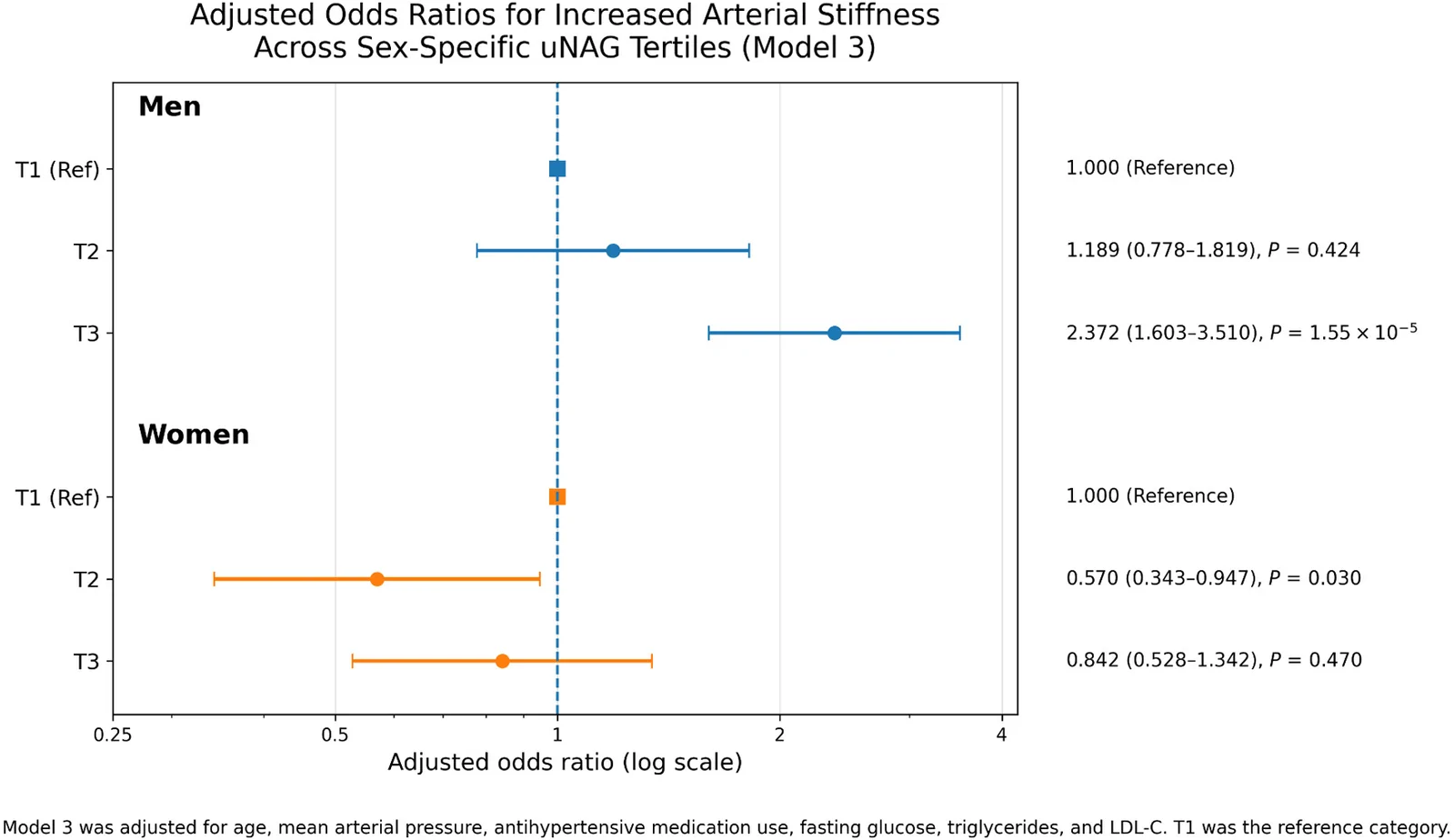
Multivariable-adjusted odds ratios for increased arterial stiffness according to sex-specific urinary N-acetyl-*β*-D-glucosaminidase tertiles. Points and horizontal lines represent adjusted odds ratios and 95% confidence intervals, respectively. The lowest sex-specific uNAG tertile (T1) was used as the reference category. Model 3 was adjusted for age, mean arterial pressure, antihypertensive medication use, fasting glucose, triglycerides, and LDL-C. In men, the adjusted odds ratios were 1.189 (95% CI, 0.778–1.819; *P* = 0.424) for T2 and 2.372 (95% CI, 1.603–3.510; *P* = 1.55 × 10⁻⁵) for T3. In women, the corresponding odds ratios were 0.570 (95% CI, 0.343–0.947; *P* = 0.030) and 0.842 (95% CI, 0.528–1.342; *P* = 0.470).

The present findings support our primary hypothesis that the association between uNAG and arterial stiffness differs by sex. The significant sex-by-uNAG interaction provides formal statistical evidence that this association differs between men and women. This conclusion is based on direct comparison of the exposure effects across sexes rather than on differences in statistical significance between separate subgroup analyses. Nevertheless, the interaction finding does not identify the biological mechanism responsible for this difference.

Several factors may contribute to the observed sex-specific association between uNAG and arterial stiffness. Men and women may differ in the distribution and accumulation of cardiometabolic risk factors, body composition, renal physiology, vascular remodeling, inflammatory activity, and treatment patterns. These differences could modify the relationship between renal tubular stress and systemic arterial stiffness. However, because the present study did not include direct measurements of menopausal status, sex hormones, body fat distribution, or inflammatory biomarkers, the biological mechanisms underlying the observed sex differences cannot be determined from our data.

In women, the absence of a consistent independent association after multivariable adjustment may reflect a more heterogeneous vascular risk profile or residual confounding by unmeasured factors. Potential contributors may include differences in age, body composition, cardiometabolic characteristics, medication use, and renal physiology, as well as the reduced measurement resolution and sensitivity of tertile construction to tied uNAG values. Because menopausal status and circulating sex hormone concentrations were unavailable, the role of hormonal factors could not be evaluated and should not be inferred from the present findings.

From a clinical perspective, our findings should be regarded as hypothesis-generating rather than immediately practice-changing. Elevated uNAG may help identify individuals with a broader phenotype of renal tubular stress and cardiometabolic vascular risk, particularly among men, but it should not be considered a stand-alone screening tool for increased arterial stiffness. Further prospective studies are needed to determine whether uNAG improves risk prediction beyond conventional risk factors and whether changes in uNAG over time are associated with progression of arterial stiffness or future cardiovascular outcomes.

### 4.4 Strengths and limitations

A key strength of this study lies in its large sample size and population-based design, which enhances the generalizability of the findings. The standardized data collection procedures, including biochemical assays and validated non-invasive vascular assessments (baPWV), ensure methodological rigor and reproducibility. Furthermore, the investigation of sex-specific associations adds valuable insight into sex-related physiological variations in vascular and renal biomarkers, an area often underrepresented in epidemiological research.

However, several limitations should be acknowledged. First, the cross-sectional nature of the study precludes causal inference. Second, although the multivariable models included age, mean arterial pressure, antihypertensive medication use, fasting glucose, triglycerides, and LDL-C, residual confounding remains possible. Antihypertensive medication use was uncommon, being reported by only 12 participants, and its coefficient was therefore imprecisely estimated. In addition, renal function indices, including serum creatinine, estimated glomerular filtration rate, and albuminuria, were unavailable; consequently, we could not determine whether the observed association was independent of glomerular dysfunction or albuminuria. Third, baPWV is a non-invasive marker of arterial stiffness and does not directly measure atherosclerotic plaque burden. Fourth, menopausal status, hormone replacement therapy, and circulating sex hormone concentrations were unavailable. Therefore, the observed differences between men and women cannot be attributed specifically to estrogen or menopausal hormonal changes. Other unmeasured sex-related biological, metabolic, behavioral, and treatment factors may also have contributed. Fifth, although statistically significant associations were observed, the correlation coefficients between uNAG and baPWV were modest and should not be interpreted as indicating strong clinical predictive performance. Finally, uNAG was measured only once, and intra-individual variability over time was not assessed. Future prospective studies incorporating conventional renal function indices, menopausal status, sex hormone measurements, body composition, inflammatory biomarkers, and longitudinal vascular outcomes are needed.

An additional limitation concerns the pronounced heaping of uNAG measurements at 5.00 IU/L among women. Although 5.00 IU/L was not used as a uniform censoring floor in the analytic dataset, as measurements below this value were present, 547 of 1,145 women (47.8%) had a recorded value of exactly 5.00 IU/L. The retrospective database did not retain the original instrument-level output, analyzer flags, detailed assay documentation, or laboratory reporting metadata required to independently verify the assay-level lower limit of quantification or determine whether the clustering reflected reporting precision, laboratory information-system conventions, rounding, or another reporting process. This reduced measurement resolution may have attenuated or otherwise influenced the continuous correlation estimate and made the female tertile classification particularly sensitive to the handling of tied values. Accordingly, the female correlation and tertile-specific estimates, particularly the intermediate-tertile finding, should be interpreted cautiously and should not be considered evidence of a precise continuous dose-response relationship, a biological threshold, or a nonlinear protective association.

## 5 Conclusion

In conclusion, higher uNAG levels were statistically associated with increased arterial stiffness, as assessed by baPWV, with a stronger independent association observed in men. However, given the modest effect sizes and the cross-sectional design, the clinical implications of these findings should be interpreted cautiously. Future longitudinal studies are required to determine whether uNAG provides incremental predictive value for vascular aging or cardiovascular events beyond established risk factors.

## Data Availability

The raw data supporting the conclusions of this article will be made available by the authors, without undue reservation.
